# Immersive motor–cognitive virtual reality training for cognitive frailty: a systematic review and meta-analysis of randomized controlled trials

**DOI:** 10.3389/fspor.2026.1754944

**Published:** 2026-04-07

**Authors:** Sensen Pan, Bo Zheng, Feifei Zhang, Liyan Lin

**Affiliations:** 1 Department of Rehabilitation, The Second Affiliated Hospital and Yuying Children’s Hospital of WMU, Zhejiang, China; 2 Global Visiting Scholar Program, UNC of Greensboro, Greensboro, NC, United States

**Keywords:** cognitive frailty, immersive virtual reality, meta-analysis, physical frailty, randomized controlled trials (RCTs)

## Abstract

**Background:**

Cognitive frailty—defined as the coexistence of physical frailty and mild cognitive impairment without dementia—has gained attention as a potentially reversible condition linked to functional decline, disability, and future dementia. Traditional motor–cognitive or physical training programs can be beneficial, yet they often suffer from low engagement and limited ecological relevance. Immersive virtual reality (VR) has emerged as a promising alternative because it provides interactive, multisensory environments capable of simultaneously stimulating cognitive and motor processes. Although several trials have evaluated VR in older adults with cognitive frailty, the overall effect remains uncertain. Recent meta-analyses have generally been broad in scope, combining heterogeneous populations or non-immersive VR systems, and no review has specifically focused on immersive motor–cognitive VR in individuals formally diagnosed with cognitive frailty.

**Objective:**

To evaluate whether immersive motor–cognitive VR improves global cognition and physical frailty in adults with cognitive frailty.

**Methods:**

Five databases were searched through March 09, 2026, for randomized controlled trials using immersive or semi-immersive motor–cognitive VR in adults diagnosed with cognitive frailty. Global cognition was the primary outcome and physical frailty the secondary. Pooled effect sizes were calculated using SMDs or MDs with 95% CIs. Risk of bias was assessed with Cochrane criteria, and certainty of evidence with GRADE.

**Results:**

Three randomized controlled trials (*n* = 344) comparing VR-based interventions with non-VR control conditions demonstrated a significant improvement in global cognitive function (SMD = 0.50; 95% CI 0.14–0.85; *I*^2^ = 39%). Two trials (*n* = 278) that used whole-body VR reported reductions in frailty severity (MD = −0.26; 95% CI −0.47 to −0.04; *I*^2^ = 0%).

**Conclusion:**

Immersive motor–cognitive VR may improve both cognitive function and frailty severity in adults with cognitive frailty. Evidence remains limited by the small number of trials and variation in VR systems and intervention protocols. Larger, high-quality studies are needed to confirm these findings

**Systematic Trial Registration:**

https://www.crd.york.ac.uk/PROSPERO/view/CRD420251234169, PROSPERO database CRD420251234169.

## 1 Introduction

Cognitive frailty is increasingly recognized as a clinically important and potentially reversible geriatric syndrome and identifying effective interventions for this population remains an important research priority (1).

In recent years, virtual reality (VR) has gained attention as a non-pharmacological option for adults with cognitive impairment. By creating immersive and interactive multisensory settings, VR can engage both cognitive functions—such as executive skills, memory, and attention—and physical abilities, including balance, gait, and strength. This game-like approach may help address common challenges seen with conventional cognitive or exercise programs, for example boredom, low motivation, or limited access to appropriate environments. From a mechanistic perspective, VR-based training may promote neuroplasticity by providing enriched sensory input, facilitating sensorimotor integration, and combining cognitive and motor tasks within dual-task environments. These mechanisms may support adaptive neural changes in brain regions associated with executive control, attention, and motor planning. In addition, a recent systematic review and meta-analysis reported that VR-based interventions significantly improved executive function in older adults with mild cognitive impairment, further supporting the potential of immersive VR training to target higher-order cognitive control processes relevant to dual-task performance (2). Evidence from recent systematic reviews and meta-analyses suggests that immersive or interactive VR interventions can improve cognitive performance, executive function, and gait-related outcomes in older adults with cognitive impairment or mild cognitive impairment (MCI) (3). In addition, VR-based exergaming systems may enhance motivation and adherence to training programs while encouraging movement within a controlled and safe virtual environment (4).

Epidemiologic research and early pilot trials have suggested that exposure to virtual reality may improve global cognition, specific cognitive abilities, and indicators of physical frailty in adults with either mild cognitive impairment (MCI) or physical frailty considered separately. For example, immersive and semi-immersive VR programs have shown advantages in domains such as executive function, attention, and balance among people with MCI, and have been associated with improvements in frailty status among physically frail individuals (3). However, when focusing on cognitive frailty—a condition requiring both cognitive deficits and physical frailty—the available evidence remains sparse and varies in quality. Some randomized controlled trials (RCTs) report meaningful gains in cognitive outcomes and frailty indices, whereas others indicate only modest or domain-limited effects (5).

Despite growing interest in this area, existing meta-analyses have several limitations that hinder their application to cognitively frail populations. Many reviews focus solely on MCI or physical frailty and do not apply the formal diagnostic framework for cognitive frailty, which generally refers to the coexistence of physical frailty or prefrailty and cognitive impairment without dementia, although operational definitions vary across studies. Notably, the latest meta-analysis published in 2025 combined VR trials across a broad range of “cognitive impairment” without identifying cognitive frailty as a separate syndrome (5). Consequently, its pooled effects were derived from heterogeneous samples that did not uniformly meet established CF diagnostic criteria, reducing the relevance of the findings for this dual-domain condition.

A further concern involves the wide variety of VR systems included in earlier analyses. These range from non-immersive screen-based interfaces to low-engagement exergaming platforms, rather than focusing specifically on immersive or semi-immersive VR capable of delivering coordinated motor–cognitive stimulation (6). This is problematic because mechanisms believed to be especially important for cognitive frailty—such as motor–cognitive integration, stepping-based training, and high sensory immersion—are not consistently provided across all VR modalities. Earlier reviews also rarely incorporated validated frailty measures such as the Fried phenotype and often aggregated cognitive assessments without harmonizing widely used global measures like the MoCA or MMSE. Trials centered on upper-limb ADL-focused VR or non-exercise VR were sometimes pooled with motor–cognitive VR interventions, adding to outcome heterogeneity and obscuring treatment effects.

These methodological gaps support the need for a more targeted meta-analysis that (1) includes only adults diagnosed with cognitive frailty using standardized definitions, (2) synthesizes evidence from immersive or semi-immersive VR interventions designed to deliver simultaneous motor–cognitive engagement, and (3) evaluates outcomes using consistent global cognitive and validated frailty measures. With these considerations, the current review aims to provide the first CF-specific quantitative synthesis of VR-based motor–cognitive interventions.

Despite growing interest in virtual reality–based interventions for older adults with cognitive impairment or frailty, important gaps remain in the literature regarding their application to cognitive frailty. Most previous systematic reviews and meta-analyses have focused on populations with mild cognitive impairment or physical frailty alone rather than individuals with cognitive frailty, a condition characterized by the coexistence of cognitive impairment and physical frailty. Furthermore, earlier reviews frequently combined heterogeneous VR modalities, including non-immersive screen-based interfaces and low-engagement exergaming systems, which may not provide the integrated motor–cognitive stimulation necessary for addressing cognitive frailty. In addition, validated frailty measures such as the Fried Frailty Phenotype and standardized global cognitive assessments (e.g., MoCA or MMSE) have not always been consistently applied in previous syntheses. Consequently, the effectiveness of immersive motor–cognitive VR interventions specifically for adults with cognitive frailty remains unclear. Therefore, the present systematic review and meta-analysis aimed to synthesize evidence from randomized controlled trials evaluating immersive or semi-immersive motor–cognitive VR interventions in adults diagnosed with cognitive frailty and to assess their effects on global cognition and physical frailty.

## 2 Methods

### 2.1 Eligibility criteria

This review was conducted in accordance with the PRISMA 2020 statement for reporting systematic reviews and meta-analyses, which served as a reporting checklist, and the study protocol was prospectively registered in the PROSPERO database (registration number: CRD420251234169).

Eligibility criteria were defined using the PICOS framework. The target population comprised adults diagnosed with cognitive frailty.

In the intervention group, participants received immersive or semi-immersive virtual reality interventions targeting cognitive function, with or without concurrent motor engagement. For the physical frailty analysis, only interventions involving substantial whole-body motor engagement were eligible. Non-immersive screen-based VR systems without interactive engagement were excluded. In the control group, participants received usual care, conventional physical or cognitive training, or no intervention. The primary outcome was global cognitive function, measured using the MoCA, Mini-Mental State Examination (MMSE), or equivalent tools. Secondary outcomes included physical frailty, assessed by the Fried Frailty Phenotype (score or reversal rate).

The inclusion criteria were as follows: (1) Study design: Randomized controlled trials (RCTs) evaluating the effects of VR-based interventions compared with control conditions in adults diagnosed with cognitive frailty. (2) Participants: Individuals diagnosed with cognitive frailty using validated operational criteria, defined as the simultaneous presence of physical frailty (Fried Frailty Phenotype score ≥1) and cognitive impairment (typically indicated by a Clinical Dementia Rating of 0.5; MoCA scores ≤25 were accepted as a commonly used screening cutoff in prior CF studies), in the absence of dementia. (3) Intervention: Immersive or semi-immersive VR interventions targeting cognitive function, with or without concurrent motor engagement, were eligible for the cognitive outcome analysis. For the physical frailty analysis, only interventions involving substantial whole-body motor engagement were included (e.g., multi-directional stepping, reaching, squatting, weight-shifting, or dual-task walking). Seated, upper-limb–dominant, non-immersive, or cycling-only VR systems were excluded. (4) Control: Participants in the control group received usual care, waiting-list control, conventional cognitive training, or non-VR motor–cognitive activities. Control conditions involving structured whole-body physical exercise (e.g., treadmill-only training, cycling-only exercise, or resistance-training programs) were excluded. (5) Other criteria: No restrictions were placed on publication year, language, VR device type, intervention duration, or follow-up length.

Studies were excluded if they met any of the following conditions: (1) Control group primarily delivered structured whole-body physical exercise programs (e.g., resistance training, treadmill-only programs, or cycling-only exercise). This criterion was applied to ensure that the comparison focused on VR-based motor–cognitive interventions vs. usual care or conventional cognitive or low-intensity activities, rather than comparing VR with structured physical exercise programs that may independently produce substantial improvements in physical function and frailty outcomes. (2) VR systems used solely for assessment purposes rather than as an intervention. (3) Unpublished studies, including conference abstracts, study protocols, and other gray literature without full-text results. (4) Studies lacking original research data or duplicate publications. The protocol for this systematic review and meta-analysis was prospectively registered in the PROSPERO database.

### 2.2 Search strategy

Literature was systematically searched in five databases (EMBASE, PubMed, Cochrane Library, Web of Science, and Scopus). Following the peer reviewers' comments, the search strategy was refined, and the literature search was repeated, updating the results through March 09, 2026. The search strategy was developed and reported in accordance with the PRISMA-S (Preferred Reporting Items for Systematic Reviews and Meta-Analyses Search Extension) guidelines.

The search strategy combined controlled vocabulary and free-text keywords related to three main concepts: (1) cognitive frailty, (2) virtual reality, and (3) randomized studies. For cognitive frailty, the following terms were used: frailty AND cognitive, cognitive frailty, and cognitively frail. For the virtual reality concept, both Medical Subject Headings (MeSH) and text words were applied, including Virtual Reality [MeSH], virtual reality, immersive, head-mounted display, and virtual environment. For study design, terms related to randomized studies were used, including random* and trial. Boolean operators (AND/OR) were used to combine the three concepts. The PubMed search strategy was constructed as follows: ((((frailty[Title/Abstract]) AND (cognitive[Title/Abstract])) OR ((cognitive frailty[Title/Abstract]) OR (cognitively frail[Title/Abstract]))) AND (((((Virtual Reality[MeSH Terms]) OR (Virtual Reality[Title/Abstract])) OR (immersive[Title/Abstract])) OR (head-mounted display*[Title/Abstract])) OR (virtual environment*[Title/Abstract]))) AND ((random*[Title/Abstract]) OR (trial[Title/Abstract])). No restrictions were applied to publication status or language. Clinical trial registries were not searched because the review focused on published randomized controlled trials with available outcome data. The search strategy was initially developed in PubMed and subsequently adapted to the syntax and indexing systems of the other databases. The full search strategies for all databases are provided in Supplementary Appendix S1.

### 2.3 Study selection

Two investigators independently reviewed the titles and abstracts of all identified records. Full-text articles were then examined to determine eligibility for inclusion. Any discrepancies during the selection process were resolved through discussion and, when necessary, by consultation with a third reviewer.

### 2.4 Data extraction

Relevant study characteristics—including the first author's name, publication year, country, group (VR-based intervention vs. control), sample size (*N*), participants' mean age, BMI (kg/m^2^), percentage or number of males, prior VR experience (*n*), training duration, baseline global cognitive function (MoCA or MMSE score), baseline physical frailty (Fried Frailty Phenotype score), effect sizes (SMD for global cognition and frailty where reported or calculable), and randomization method—were systematically extracted from each eligible randomized controlled trial.

Data extraction was performed independently by two reviewers, and discrepancies were resolved through discussion or consultation with a third reviewer. When essential details were missing or unclear (e.g., exact SD for effect sizes or missing baseline values), attempts were made to contact the corresponding authors for clarification or additional information.

### 2.5 Study risk of bias assessment

The methodological quality of each included study was evaluated independently by two reviewers using Review Manager (RevMan) version 5.4. The assessment covered key domains of bias, including random sequence generation, allocation concealment, blinding, incomplete outcome data, selective reporting, and other potential sources of bias. Each domain was rated as having a high, low, or unclear risk of bias according to Cochrane criteria.

### 2.6 Data synthesis

All statistical analyses were conducted using RevMan version 5.4. A two-sided *P* value ≤0.05 was considered statistically significant. Because different cognitive assessment tools were used across studies (MoCA and MMSE), standardized mean differences (SMDs) were calculated to ensure comparability. For physical frailty outcomes measured using the same scale (Fried Frailty Phenotype), mean differences (MDs) were used. Effect sizes were calculated using the change from baseline to post-intervention. When studies did not report the standard deviation of the change scores, it was calculated using baseline and post-intervention standard deviations according to the Cochrane Handbook approach, assuming a correlation coefficient of 0.5 between baseline and post-intervention measurements. Pooled estimates were calculated using the inverse-variance method under a random-effects model. Statistical heterogeneity was assessed using the *I*^2^ statistic, with values of <25%, 26%–50%, and >50% representing low, moderate, and high heterogeneity, respectively. Only change-from-baseline values were used in the quantitative synthesis.

### 2.7 Assessment of publication bias

Evaluation of publication bias was not performed because fewer than ten studies were included, which limits the statistical power of funnel plot analysis to detect asymmetry. Nevertheless, it should be recognized that the small number of available trials may predispose the findings to potential publication bias (7).

## 3 Results

### 3.1 Studies selection

**Figure 1:**
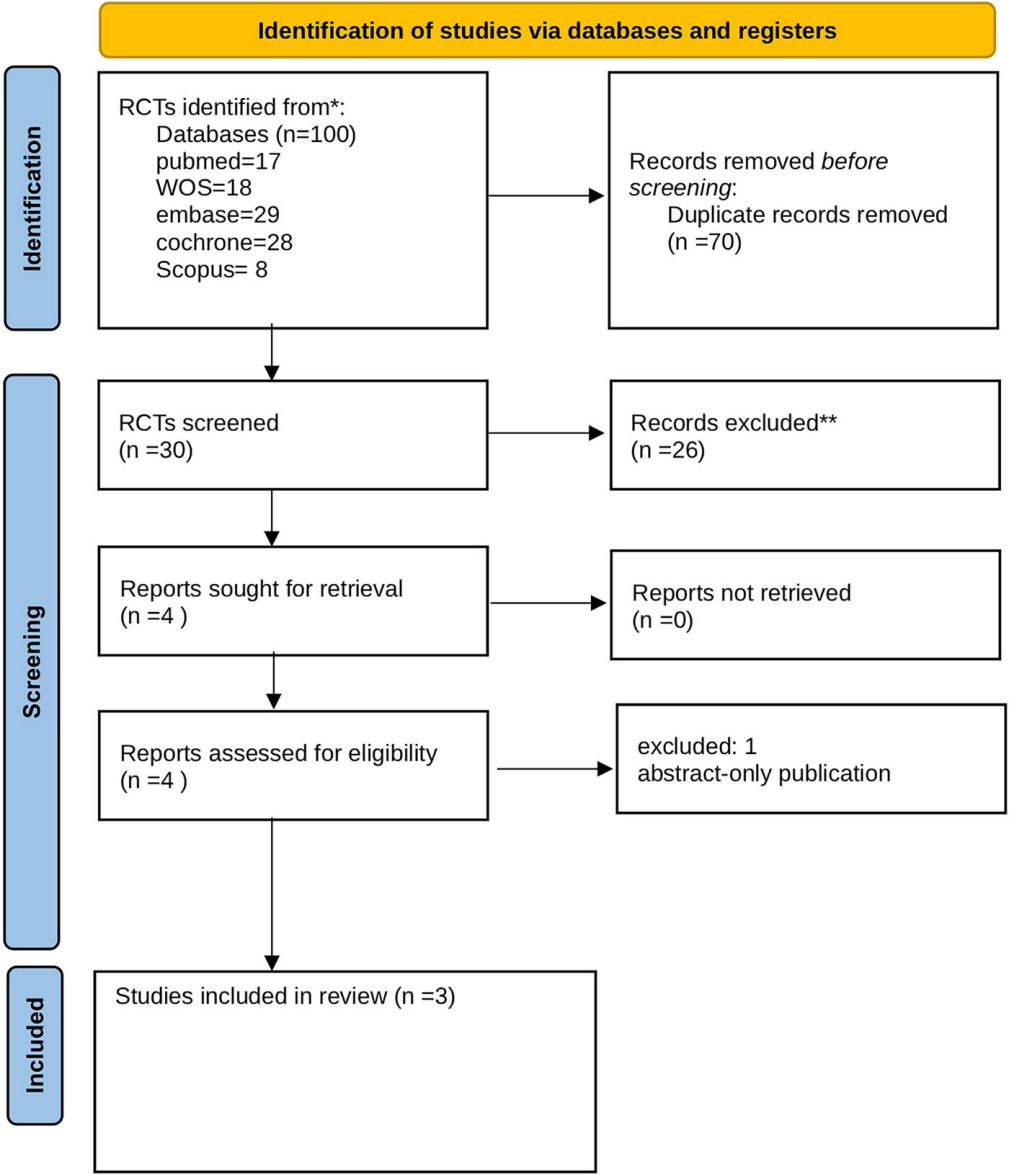
The PRISMA flow diagram. PRISMA, preferred reporting items for systematic reviews and meta-analyses.

A total of 100 records were initially identified through database searching (PubMed *n* = 17, Web of Science *n* = 18, Embase *n* = 29, Cochrane Library *n* = 28, Scopus *n* = 8). After removing 70 duplicates, 30 records were screened based on title and abstract, of which 26 were excluded. Four reports were sought for retrieval and subsequently assessed for eligibility; one was excluded because it was an abstract-only publication. Ultimately, three studies met the eligibility criteria and were included in the systematic review and meta-analysis. The detailed process of study selection is presented in the PRISMA 2020 flow diagram (Figure 1).

**Figure 2:**
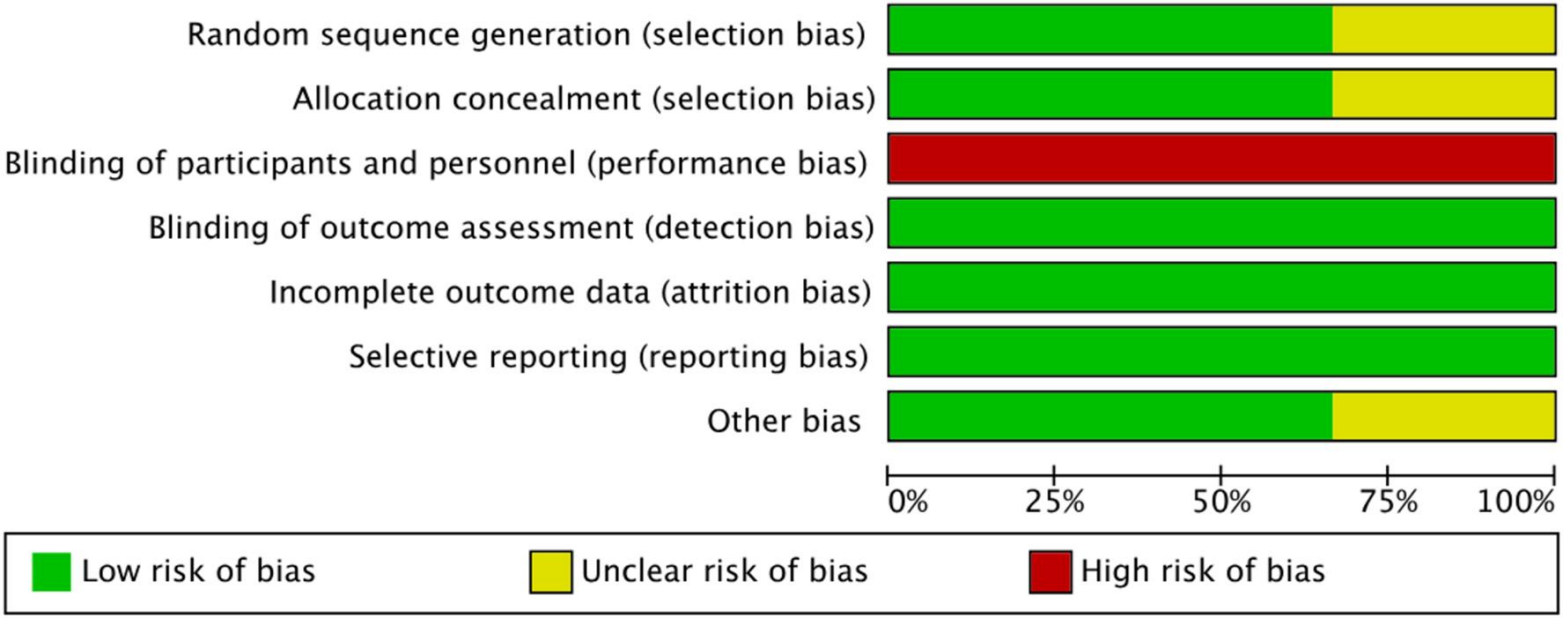
Risk of bias graph.

**Figure 3:**
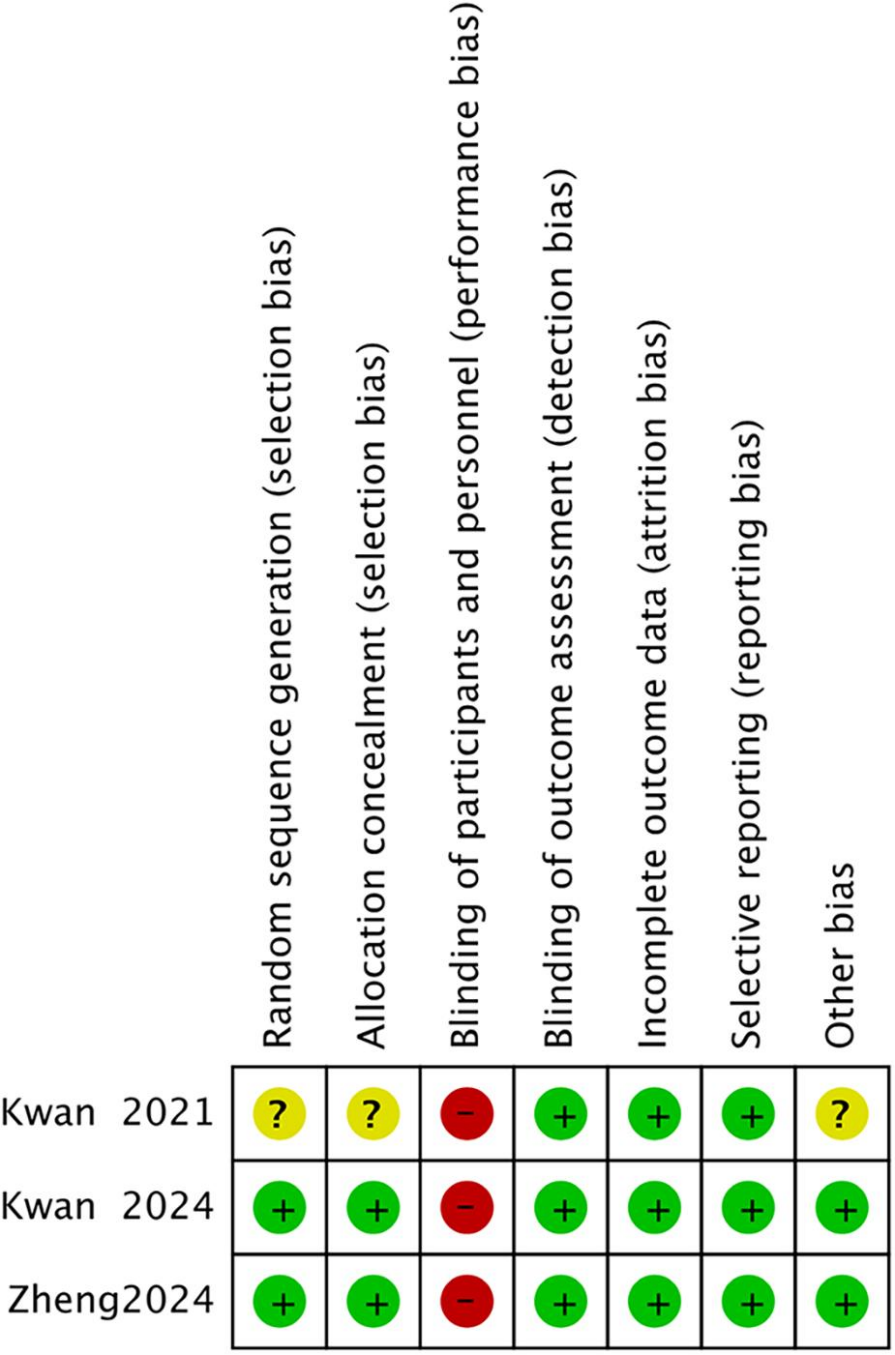
Risk of bias summary.

### 3.2 Characteristics of included studies

A total of three randomized controlled trials were included in this meta-analysis. All three studies were conducted in China [two in Hong Kong (4, 8), and one in mainland China (9)]. The total sample size across all trials was 344 participants (VR-based intervention groups: *n* = 167; control groups: *n* = 177), with individual study sizes ranging from 17 participants per group in the two smaller trials to 125–136 in the largest trial. Participant mean ages ranged from 73.9 to 80.9 years. All participants met diagnostic criteria for cognitive frailty, which includes physical frailty and cognitive impairment. Baseline global cognitive status was impaired (MoCA typically 19–23 or equivalent on MMSE), and most participants were frail according to the Fried Frailty Phenotype.

Two trials delivered VR-based multi-domain motor–cognitive training with substantial whole-body physical engagement (4, 8), whereas the third trial employed primarily cognitive VR tasks without whole-body involvement (9). Therefore, the third trial was not included in the physical frailty meta-analysis. Intervention duration was 8 weeks in two trials and 12 weeks in one trial.

The primary outcomes of interest in the current meta-analysis were changes in global cognitive function measured by MoCA and/or MMSE, with additional data on Fried Frailty Phenotype where reported. Dropout rates were low and generally balanced between groups. Most participants had little or no prior VR experience. Randomization methods were adequately reported in two trials (8, 9), whereas the pilot trial provided insufficient detail (4). The characteristics and extracted data of all included studies are summarized in Tables 1, 2.

**Table 1:** Main characteristics of the included randomized controlled trials evaluating VR-based motor–cognitive training vs. non-VR control conditions in older adults diagnosed with cognitive frailty.

Study ID	Year	Country	Group	*N*	Age	BMI (kg/m^2^)	Male (*n*)	Training duration (weeks)	VR experience (*n*)	Change in MoCA (mean ± SD)	Change in MMSE (mean ± SD)	Effect size for global cognition (SMD ± SD) (MoCA and MMSE)	Change in fried frailty phenotype	Randomization method
Kwan	2021	China (Hong Kong)	VRMCT	9	73	24.4 ± 6.3	1	8	0	4.00 ± 3.29	-	1.216 ± 1.35	−1.00 ± 0.74	Excel-generated random list
			MCT	8	77.5	22.2 ± 2.6	1	8	0	2.00 ± 3.33	-	0.601 ± 1.10	−1.00 ± 1.16	
Kwan^b^	2024	China (Hong Kong)	VRMCT	125	75.2 ± 7.1	24.4 ± 3.7	36	8	18	1.00 ± 2.96	-	0.338 ± 1.03	−0.31 ± 0.81	Unclear
			Usual care	136	73.9 ± 6.6	24.2 ± 3.5	28	8	42	0.04 ± 3.19	-	0.013 ± 1.00	−0.04 ± 1.00	
Zheng	2025	China	VR	33	80.88 ± 8.75	24.16 ± 3.28	12	12	-	-	2.43 ± 9.01	0.270 ± 1.02	−1 ± 0.72^a^	Computer-generated randomization list
			usual care	33	79.52 ± 9.61	23.03 ± 3.20	18	12	-	-	−2 ± 3.18	−0.629 ± 1.10	0 ± 0.89^a^	

VRMCT, Virtual reality motor-cognitive training; VR, virtual reality; MCT, motor-cognitive training.

^a^ Excluded from fried frailty phenotype because the intervention group didn't receive whole-body engagement.

^b^ 21 participants in the VRMCT group and 11 in the control group were lost to follow-up; therefore, the analyzed sample sizes were 125 and 136, respectively.

**Table 2:** Characteristics of VR-based interventions.

Study	Frequency (sessions/week)	Session duration (min)	Intervention period (weeks)	Total sessions	VR modality	Control condition
Kwan 2021	2	30	8	16	Immersive VR simultaneous motor–cognitive training using head-mounted display and cycling ergometer	Non-VR sequential motor–cognitive training
Kwan 2024	2	60	8	16	Immersive VR motor–cognitive training (VRMCT) using VR headset and motion-sensor bicycle ergometer	Usual care
Zheng 2025	2	45	12	24	Immersive VR-based ADL rehabilitation training using head-mounted display and controllers	Usual care

### 3.3 Risk of biases assessment

The visual representation of bias assessment is shown as a risk of bias graph (Figure 2) and a table (Figure 3). The risk of bias of the included randomized controlled trials was evaluated using the Cochrane Risk of Bias Tool version 1 (RoB 1). Overall, the methodological quality of the included studies was acceptable. Two large trials (8, 9) demonstrated generally low risks of bias, with adequate random sequence generation and allocation concealment, as both studies used computer-generated randomization lists and sealed opaque envelopes for allocation. Outcome assessors were blinded in all trials. However, blinding of participants and personnel was not feasible due to the nature of VR-based interventions; therefore, all studies were rated as having a high risk of performance bias. A small pilot RCT (4), provided insufficient information on the methods of random sequence generation and allocation concealment, resulting in unclear risks for these domains. Despite the small sample size, attrition was minimal and well explained across all studies. All trials reported outcomes according to their prespecified protocols or trial registrations, indicating a low risk of selective reporting. No significant concerns were identified regarding incomplete outcome data or other potential sources of bias in the larger trials. In summary, two full-scale RCTs showed overall low risks of bias except for performance bias, whereas the pilot RCT showed higher uncertainty due to limited methodological reporting.

### 3.4 Primary outcomes

**Figure 4:**
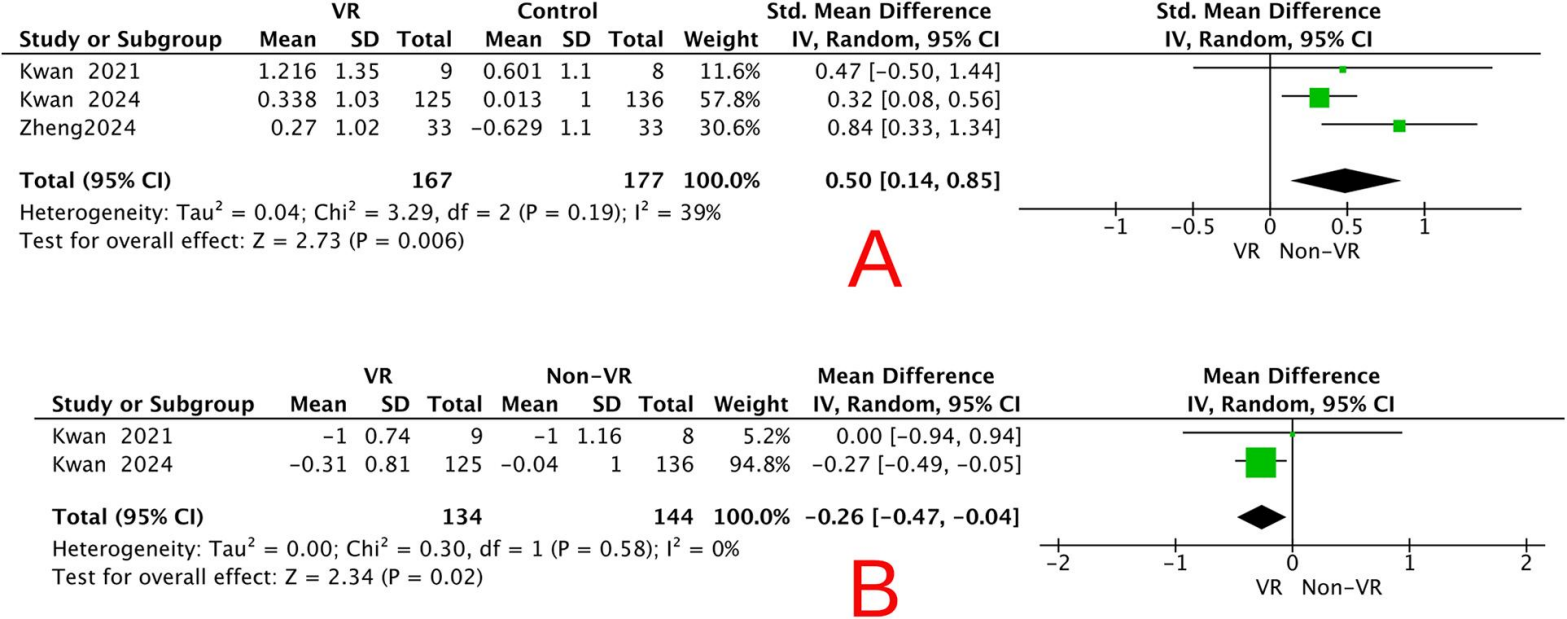
Forest plots showing the pooled effects of VR-based interventions compared with non-VR control conditions in older adults with cognitive frailty. **(A)** Global cognitive function measured using MoCA or MMSE (standardized mean difference). **(B)** Physical frailty measured using the Fried Frailty Phenotype (mean difference).

Three randomized controlled trials (total *n* = 344) evaluated the effects of virtual reality (VR)–based motor–cognitive training on global cognitive function (assessed by MoCA and/or MMSE) in older adults diagnosed with cognitive frailty. Pooled analysis indicated that VR-based motor–cognitive training was associated with a modest improvement in global cognitive function compared with non-VR control conditions [standardized mean difference (SMD) = 0.50, 95% CI 0.14–0.85, *P* = 0.006] (Figure 4A). Heterogeneity across studies was low to moderate (*P* = 0.19; *I*^2^ = 39%), suggesting reasonable consistency among the included trials.

### 3.5 Secondary outcomes

Two randomized controlled trials (total *n* = 278) examined the effects of VR-based motor–cognitive training on physical frailty, as measured by the Fried Frailty Phenotype, in older adults diagnosed with cognitive frailty. Pooled analysis indicated that VR-based motor–cognitive intervention was associated with a modest reduction in frailty severity compared with non-VR control conditions [mean difference (MD) = −0.26, 95% CI −0.47 to −0.04; *Z* = 2.34, *P* = 0.02] (Figure 4B). Heterogeneity between the two studies was negligible (*P* = 0.58; *I*^2^ = 0%), indicating excellent consistency. Because only two studies were available for this outcome, the results should be interpreted cautiously despite the low statistical heterogeneity.

### 3.6 GRADE level

According to the GRADE assessment, the certainty of the evidence was low for both primary outcomes. For global cognitive function (MoCA/MMSE), the evidence was downgraded one level due to unclear randomization methods in one trial and unavoidable performance bias related to the lack of blinding of participants and personnel and downgraded one additional level for imprecision because of the small number of included studies and the relatively wide confidence interval. No serious concerns were identified regarding inconsistency, indirectness, or publication bias. For physical frailty (Fried Frailty Phenotype), the evidence was similarly downgraded one level for the same methodological limitations. However, despite the absence of statistical heterogeneity, the small number of studies and limited sample size warranted downgrading for imprecision. This meta-analysis suggests that VR-based motor–cognitive training may improve global cognitive function and reduce frailty severity in adults with cognitive frailty. However, confidence in these findings is limited by the small number of trials and their restriction to a single geographic region. These findings are promising but require confirmation in future large-scale, high-quality trials. The GRADE level is summarized in Table 3.

**Table 3:** GRADE assessment of the certainty of evidence for the included outcomes.

Outcomes	Study limitation	Imprecision	Inconsistency	Indirectness	Publication bias	GRADE
Global cognitive function	Downgraded due to unclear randomization methods in some studies.	Downgraded due to small number of trials and wide CI	No downgrade	No downgrade	No downgrade	Low
Physical frailty (fried frailty phenotype)	Downgraded due to unclear randomization methods in some studies.	Downgraded due to only two trials and small sample size	No downgrade	No downgrade	No downgrade	Low

## 4 Discussion

Because cognitive frailty is a relatively new clinical concept and immersive VR technology has only recently begun to be incorporated into geriatric research, it was anticipated that few RCTs would meet our stringent inclusion standards. To date, only three studies worldwide have used a validated diagnostic algorithm for cognitive frailty, applied immersive or semi-immersive VR systems, and adopted a randomized controlled design. The small number of eligible trials therefore reflects the early stage of development in this research area rather than any shortcomings in the search process. A similar challenge has been observed in other emerging clinical fields where only a limited number of RCTs are available for quantitative synthesis (10).

Across these three RCTs, which together enrolled 344 older adults with cognitive frailty, VR-based motor–cognitive interventions produced a statistically significant improvement of moderate magnitude in global cognitive performance (SMD = 0.50, 95% CI 0.14–0.85; *Z* = 2.73, *P* = 0.006). A particularly interesting observation was the simultaneous reduction in frailty severity reported in the two studies that evaluated the Fried Frailty Phenotype. Although the pooled effect size for global cognition was in the moderate range, its clinical relevance should be interpreted cautiously because only a small number of trials were available and the durability of the observed benefit remains unknown.

Heterogeneity for the primary cognitive outcome was low to moderate (*I*^2^ = 39%). This variation appears to stem mainly from differences in the comparison groups—two trials from Kwan and colleagues used active, multi-domain cognitive training as control conditions, whereas the mainland China study implemented a passive educational video control—and from small differences in intervention length (8 vs. 12 weeks). The most recent and largest trial carried substantial weight in the pooled analysis, helping to stabilize the overall effect size. In contrast, heterogeneity for frailty outcomes was essentially absent (*I*^2^ = 0%), reflecting the highly consistent findings across both trials using similar VR motor–cognitive protocols. The intervention characteristics across the included trials were also relatively consistent. As summarized in Table 2, all studies delivered VR-based training twice per week, with session durations ranging from 30 to 60 min and intervention periods ranging from 8 to 12 weeks. This relatively homogeneous intervention schedule may partly explain the comparable direction of effects observed across studies and suggests that a moderate and feasible training dose may be sufficient to produce measurable benefits in older adults with cognitive frailty.

The observed improvement in frailty is noteworthy, although these VR programs were not designed as conventional exercise interventions. Several mechanisms may contribute to this dual benefit. Immersive VR platforms used in these trials—such as systems based on Xbox Kinect or full-body motion tracking—require users to perform physically demanding movements, including stepping, reaching, squatting, and dual-task walking. As a result, participants may receive additional physical stimulation through movement-based tasks. The gamified design of VR interventions may enhance participant engagement compared with conventional cognitive or computer-based tasks. Gains in executive control, attention, and motor planning achieved through motor–cognitive VR may also translate into better functional mobility and confidence, influencing frailty components such as gait speed and grip strength (4, 8, 9).

These findings are consistent with a broader body of research showing that integrated cognitive–motor training approaches—including dual-task VR and exergaming—tend to outperform single-domain cognitive or physical programs in improving physical performance and reducing frailty and fall risk in older adults. Recent meta-analyses of virtual reality interventions in older adults with cognitive impairment have reported similar findings. For instance, previous systematic reviews have shown that VR-based training can produce small-to-moderate improvements in global cognitive performance and executive function compared with conventional cognitive training. These studies suggest that the immersive and interactive characteristics of VR may enhance engagement and promote simultaneous stimulation of cognitive and motor processes (5, 11).

### 4.1 Limitations

Several limitations of the present review should be acknowledged. First, only three trials met the inclusion criteria, all conducted in East Asia (Hong Kong and mainland China), which may limit the external validity of the findings. Second, intervention durations were relatively short (8–12 weeks), providing no insight into the long-term sustainability or potential disease-modifying effects of VR-based training. Third, blinding of participants and personnel was inherently challenging in behavioral VR interventions, and the pilot trial provided insufficient detail on random sequence generation and allocation concealment, introducing potential risk of selection bias. Finally, although dropout rates were low and adverse events were minimal—predominantly mild cybersickness that resolved quickly—the long-term safety of immersive VR in frailer individuals remains uncertain.

Despite these limitations, this meta-analysis provides low-certainty evidence that short-term VR-based motor–cognitive training improves global cognitive performance and reduces physical frailty in older adults diagnosed with cognitive frailty. The dual benefit, achieved through a single engaging intervention, highlights VR as a promising, scalable, and enjoyable therapeutic modality for this vulnerable population. Future research should prioritize large, multicenter, longer-duration trials with active physical comparators, domain-specific cognitive outcomes, and extended follow-up to confirm durability and establish optimal dosing and implementation strategies. Taken together, the present findings support the potential role of immersive VR-based motor–cognitive interventions as a promising non-pharmacological strategy for addressing the dual impairments of cognition and frailty in aging populations.

## 5 Conclusion

VR-based motor–cognitive interventions appear to offer meaningful gains in global cognitive performance and may also lessen the severity of physical frailty in older adults with cognitive frailty, likely owing to the simultaneous cognitive–motor demands and the high level of engagement these systems provide. Even so, the current evidence base remains small, consisting of only three studies conducted within a single geographic area and characterized by relatively short follow-up periods. To strengthen the field, larger multicenter trials with rigorous designs, longer intervention durations, and extended follow-up are needed to verify these effects, assess their durability, and identify the most effective approaches for real-world implementation.

## Data Availability

The original contributions presented in the study are included in the article/Supplementary Material, further inquiries can be directed to the corresponding author.
