# Supplementary material for: Immersive motor–cognitive virtual reality training for cognitive frailty: a systematic review and meta-analysis of randomized controlled trials
Source: Front Sports Act Living. 2026 Apr 7;8:1754944. doi: 10.3389/fspor.2026.1754944 (PMC13095833; doi:10.3389/fspor.2026.1754944)
Supplement: Supplementary file 1 [file Datasheet1.docx]

search from inception to March 09, 2026

Pubmed

((((frailty[Title/Abstract]) AND (cognitive[Title/Abstract])) OR ((cognitive frailty[Title/Abstract]) OR (cognitively frail[Title/Abstract]))) AND (((((Virtual Reality[MeSH Terms]) OR (Virtual Reality[Title/Abstract])) OR (immersive[Title/Abstract])) OR (head-mounted display*[Title/Abstract])) OR (virtual environment*[Title/Abstract]))) AND ((random*[Title/Abstract]) OR (trial[Title/Abstract]))

Scopus

TITLE-ABS-KEY ( ( random* OR trial ) AND ( "cognitive frailty" OR "cognitively frail" OR ( frailty AND cognitive ) ) AND ( "virtual reality" OR virtual environment* OR immersive OR "head-mounted display*" ) )

Embase

('virtual reality':ti,ab,kw OR 'virtual environment*':ti,ab,kw OR immersive:ti,ab,kw OR 'head-mounted display*':ti,ab,kw)

AND

(random*:ti,ab,kw OR trial:ti,ab,kw)

AND

(

('frailty':ti,ab,kw AND cognitive:ti,ab,kw)

OR

('cognitively frail':ti,ab,kw OR 'cognitive frailty':ti,ab,kw)

)

Cochrane

#1 (frailty):ti,ab,kw AND (cognitive):ti,ab,kw

OR (cognitively frail):ti,ab,kw

OR (cognitive frailty):ti,ab,kw

#2 ("virtual reality"):ti,ab,kw

OR ("head-mounted display"):ti,ab,kw

OR ("head-mounted displays"):ti,ab,kw

OR (immersive):ti,ab,kw

OR ("virtual environment"):ti,ab,kw OR ("virtual environments"):ti,ab,kw

#3 #1 AND #2

Web of Science

TS=(

(

frailty AND cognitive

)

OR

(

"cognitive frailty"

OR cognitively frail

)

)

AND

TS=(

"virtual reality"

OR immersive

OR "head-mounted display*"

OR "virtual environment*"

)

AND

TS=(

random*

OR trial

)
